# Small Molecules of Natural Origin as Potential Anti-HIV Agents: A Computational Approach

**DOI:** 10.3390/life11070722

**Published:** 2021-07-20

**Authors:** Luminita Crisan, Alina Bora

**Affiliations:** “Coriolan Dragulescu” Institute of Chemistry, 24 M. Viteazu Avenue, 300223 Timisoara, Romania

**Keywords:** natural products, HIV-1, virtual screening, docking, MM-GBSA, NNRTI

## Abstract

The human immunodeficiency virus type 1 (HIV-1), one of the leading causes of infectious death globally, generates severe damages to people’s immune systems and makes them susceptible to serious diseases. To date, there are no drugs that completely remove HIV from the body. This paper focuses on screening 224,205 natural compounds of ZINC15 NPs subset to identify those with bioactivity similar to non-nucleoside reverse transcriptase inhibitors (NNRTIs) as promising candidates to treat HIV-1. To reach the goal, an in silico approach involving 3D-similarity search, ADMETox, HIV protein-inhibitor prediction, docking, and MM-GBSA free-binding energies was trained. The FDA-approved HIV drugs, efavirenz, etravirine, rilpivirine, and doravirine, were used as queries. The prioritized compounds were subjected to ADMETox, docking, and MM-GBSA studies against HIV-1 reverse transcriptase (RT). Lys101, Tyr181, Tyr188, Trp229, and Tyr318 residues and free-binding energies have proved that ligands can stably bind to HIV-1 RT. Three natural products (ZINC37538901, ZINC38321654, and ZINC67912677) containing oxan and oxolan rings with hydroxyl substituents and one (ZINC2103242) having 3,6,7,8-tetrahydro-2H-pyrido[1,2-a]pyrazine-1,4-dione core exhibited comparable profiles to etravirine and doravirine, with ZINC2103242 being the most promising anti-HIV candidate in terms of drug metabolism and safety profile. These findings may open new avenues to guide the rational design of novel HIV-1 NNRTIs.

## 1. Introduction

The human immunodeficiency virus type 1 (HIV-1), which causes acquired immunodeficiency syndrome (AIDS), continues to be one of the world’s most serious public health concerns. According to the World Health Organization [1], approximately 38 million people across the globe, including 36.2 million adults and 1.8 million children under 15 years, were infected with HIV/AIDS by the end of 2019. Of these, it is estimated that 68% of all people living with HIV have access to anti-retroviral therapy (ART), and 32% are still waiting. Despite advances in the scientific understanding of HIV and its treatment, 690,000 people died from HIV-related diseases in 2019. HIV-1 infection massively depletes T-cell stores by directly infecting and killing the activated CD4+ T cells, which play a key role in the immune response against infections. The steady decline of CD4+ T cells leads to an increased susceptibility to infections and eventually to the onset of AIDS [2].

The mainstay treatment of HIV infection consists of three or more antiretroviral drugs, most commonly two nucleoside reverse transcriptase inhibitors (NRTIs) together with a non-nucleoside reverse transcriptase inhibitor (NNRTIs) or a protease inhibitor (PI) or, more recently, an integrase inhibitor (INI). Independent use of NNRTI and NRTI inhibitors promotes virus resistance [3]. The current 26 approved antiretroviral drugs alongside HIV combinations medicines (FDA approved HIV medicines) do not eradicate HIV infection but suppress virus activity at different life-cycle stages. In this regard, rational drug selection is a fundamental step to decrease side effects and cross-resistance, increase potency, and prolong viral suppression. To date, ten classes of FDA-approved HIV drugs, classified by their molecular mechanism, resistance profiles, generic, and brand names, are available: (i) NRTIs, (ii) NNRTIs, (iii) INIs, (iv) PIs, (v) fusion inhibitors, (vi) CCR5 (C-C chemokine Receptor 5) antagonists, (vii) attachment and (viii) post-attachment inhibitors, (ix) pharmacokinetic enhancers, and (x) HIV medicines combination [4].

**Figure 1:**
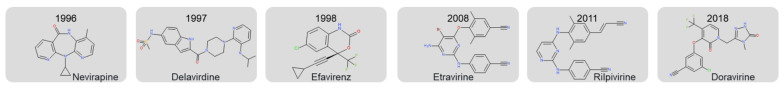
The NNRTI FDA-approved drugs for HIV-1.

The early generation of antiretroviral agents, NRTIs, is an important component of most combination therapies. Nevertheless, their uses become disadvantageous due to the large pill burden, frequent daily dosing, largely poor oral bioavailability, and severe side effects. Moreover, HIV resistance to drugs has become the main concern of this early generation. Newer designed agents, NNRTIs, an essential part of highly active antiretroviral therapy (HAART), have attracted attention due to their high specificity, strong antiviral activity, new mechanisms of action, lower pill burden or dosing frequency, and low cytotoxicity [5,6]. Etravirine (TMC125) and rilpivirine (TMC278), members of DAPY (diarylpyrimidines) family, directly bind to the HIV-1 reverse transcriptase (RT) and consequently block DNA- and RNA-dependent polymerase activity. Etravirine and rilpivirine (Figure 1) are classified as second-generation of NNRTIs. Compared to the first NNRTIs generation, nevirapine, delavirdine, and efavirenz (Figure 1), the second acquired high inhibition levels even when tested against mutated HIV-1 strains [7]. Although the first two NNRTIs generations were successful in inhibiting HIV, attempts were made to design new structures for NNRTIs future generations, such as lersivirine [8] or calanolide A [9]. However, their clinical trial evaluations did not show an improvement over existing NNRTIs, and investigations were stopped. The most recently approved NNRTI, doravirine (Figure 1), has the advantages over known NNRTIs of a new resistance pathway that preserves activity against relevant NNRTI viral mutations (K103N, Y181C, Y188L, and L100I) [10] and a more favourable drug interaction profile. Despite the advantages of doravirine over other NNRTIs, when used as a single medicine, resistance problems may occur [11]. In this context, the designs of novel candidates with enhanced pharmacokinetic properties, better activity profiles, and new therapies to combat HIV drug resistance are vital. These stringent requirements have been addressed by using various computational techniques, such as quantitative structure-activity relationships (QSAR) [12,13,14,15], pharmacophore modelling [16,17], molecular docking [15,18,19], molecular dynamics simulation [15,20,21], etc. These approaches have generated considerable interest by reducing the time required for preclinical evaluation, clinical trials, as well as costs and resources. Molecular modelling studies are valuable (i) to predict the importance of mutations for HIV-1 resistance, (ii) to explain the molecular mechanisms of resistance related to RT-ligand complex, and (iii) to reveal specific insights required to design new potent HIV-RT inhibitors [22,23,24]. However, docking approaches that portray the interactions of molecules with HIV-1 RT are not widely presented. One of the first successful examples of drug repurposing is zidovudine (AZT), originally developed as an anticancer agent and further repositioned as an anti-HIV drug [25]. Some of the anti-HIV inhibitors, ribavirin, and lopinavir in combination with ritonavir, have demonstrated antiviral potency against severe acute respiratory syndrome (SARS) and the Middle East respiratory syndrome (MERS) viruses associated with COVID-19 [26]. However, when lopinavir-ritonavir treatment was administered to adult patients with severe COVID-19, no clinical improvements beyond standard care were noticed [27].

Accordingly, the approved anti-HIV drugs could be used as (i) reference molecules to guide the design of new medicines and (ii) investigated for their re-application to other viruses or non-virus diseases using computational as well as experimental methods.

In this study, we considered the ZINC15 NPs subset of 224,205 natural compounds and screened this compounds collection using combined in silico approach against HIV-1 RT target protein to identify novel molecules possibly capable of enabling the HIV-1/AIDS management.

## 2. Materials and Methods

### 2.1. Shape-Based Virtual Screening

In the last 20 years, natural products (NPs) and their structural analogs have been considered the source of the most active ingredients in medicine as well as plying a key role in the drug discovery process. Starting from the NPs’ essential role in drug discovery, the 224,205 natural products of ZINC15 NPs subset (http://zinc15.docking.org/, accessed on 8 January 2021) [28] were downloaded and engaged in 3D shape-based similarity analysis to find similar compounds with queries molecules, efavirenz, etravirine, rilpivirine, and doravirine. A shape-based similarity search was carried out using the Rapid Overlay of Chemical Structures (ROCS) tool (OpenEye Scientific Software, Santa Fe, NM, USA) [29,30]. Usually, similarity search analysis is of interest for lead identification in drug discovery programs contributing to detection of novel chemotypes [31]. For ROCS screening, the RX conformations of the query compounds (efavirenz, etravirine, rilpivirine, and doravirine), were employed. The ZINC15 NPs subset was prepared for analysis by engendering ionization states at pH = 7.2 ± 0.2, removing salts, and generating at most 32 stereoisomers per each ligand with the aid of LigPrep (Schrödinger, LLC, New York, NY, 2018) [32]. The NPs multiple conformations were calculated using Omega (OpenEye Scientific Software, Santa Fe, NM, USA) [33,34] in the default setting of a maximum 200 conformers per compound with an energy cutoff of 10 kcal/mol relative to the global minimum identified from the search and an root-mean-square deviation (RMSD) threshold of 0.5 to remove the duplicate conformers. In ROCS, TanimotoCombo (TC), ShapeTanimoto (ShT), and ComboScore (CS) were selected as scoring parameters. Compounds that met the scoring criteria were further subjected to absorption, distribution, metabolism, excretion, and toxicity (ADMETox) evaluation; HIV protein-inhibitor prediction (HIVprotI); and docking simulations.

### 2.2. ADMETox Profile

The SwissADME [35] and pkCSM [36] online servers (accessed on 18 February 2021) were used to predict the physicochemical and pharmacokinetic parameters essential for a drug to be recommended as drug candidates. ADMETox properties prediction help medicinal chemists to rapidly design, evaluate, and prioritize a compound as a drug candidate.

### 2.3. Antiviral Activity Prediction

The antiviral activity (expressed as the half maximal inhibitory concentration, IC50, micromolar) and percent inhibition (%values) of the NPs against key HIV-1 RT enzyme were evaluated using HIVprotI platform. HIVprotI is a web-based algorithm dedicated to virtual screening and design of new inhibitors against HIV proteins, such as reverse transcriptase (RT), protease (PR), and integrase (IN). To develop this web tool, various inhibitors datasets with experimentally tested IC50/percent inhibition activity against all three HIV proteins were retrieved from the ChEMBL database and used further to develop support vector machine (SVM)-based quantitative structure activity relationship (QSAR) models employing the inhibitor features, descriptors, and fingerprints [37].

### 2.4. Molecular Docking

Molecular docking using Glide module of Schrödinger [38,39,40], was applied to predict HIV-1 RT-NPs binding modes and to rank-order NPs based GlideScore (gscore). Glide approximates the conformational space, orientation, and position of the docked ligands. The database was run through standard prediction (SP) docking mode with default options. The latter is suitable to screen a large number of ligands of unknown quality. During the docking simulation, the receptor was treated as rigid, and ligands were considered as flexible to acquire the most favorable profile of interaction with the key binding residues.

In this light, the HIV-1 RT X-ray crystal structures in complex with etravirine (PDB ID: 3MEC) [41], efavirenz (PDB ID: 1FK9) [42], and doravirine (PDB ID: 4NCG) [43] were downloaded from Protein Data Bank (http://www.rcsb.org, accessed on 31 March 2021) and further prepared and refined with the Protein Preparation Wizard [44] accessible within Maestro module of Schrodinger [45]. Summarily, the protein preparation stage involves: (i) the correction of multiple bonds, hydrogen addition, and water molecules removal beyond 5Å from the ligand; (ii) the protonation states assignment for Asp, Glu, and/or tautomers for His and the optimization of hydroxyl groups to maximize the hydrogen-bonding network; (iii) the structure postprocessing to identify and fix all the existing errors in protein structure (e.g., incomplete residues, missing side chains or loops, errors of the Asp, and Glu protonation states and tautomers of His and/or the orientation of misoriented units, such as amide unit of Asn and Gln residues); (iv) the formation of salt bridges; and (v) the protein-ligand complex refinement through a series of restrained minimizations employing the OPLS_2005 force field and an RMSD threshold value of 0.3Å [38,39,40]. Rilpivirine, the co-crystal ligand of the HIV-1 RT complex (PDB ID: 3MEE) [41], was used only for the 3D similarity search stage. The glide grid file for all X-ray complexes was created via the receptor grid generation option by selecting the co-crystallized ligand placed at the protein active site. The ligands as prepared for the shape-based virtual screening step were further used in docking simulations. The best docking pose out of five generated for each compound was selected for analysis of its ligand-target interaction. The docking protocol was validated by (i) redocking the X-ray ligand structure extracted from the crystal complex into the same binding site and (ii) calculating the RMSD between the best docked pose and corresponding X-ray ligand structure (designated as reference) using Superposition option of Maestro module of Schrödinger [45].

### 2.5. MM-GBSA Free Energies

Molecular mechanics generalized Born surface area (MM-GBSA) implemented in Prime module of Schrödinger [46,47] was employed to estimate ligand-binding affinities with potential inhibition effect on HIV-1 RT. The MM-GBSA is used to improve the shape-based virtual screening and docking results as well as to find new compounds that bind to the receptor. The ligand-receptor complexes were minimized by employing the local optimization feature of Prime with the OPLS 2005 force field. To calculate the binding free energy of the complex system, the following thermodynamic and desolvation parameters were examined: binding energy (ΔG_bind_), solvation model (ΔG_bind_ Coulomb), nonpolar solvation term (ΔG_bind_ Lipo), hydrogen-bonding correction (ΔG_bind_ Hbond), covalent binding (ΔG_bind_ Covalent), п-п packing correction (ΔG_bind_ Packing), generalized Born electrostatic solvation energy (ΔG_bind_ solv GB), and van der Waals interaction (ΔG_bind_ vdW).

## 3. Results and Discussion

### 3.1. Workflow

**Figure 2:**
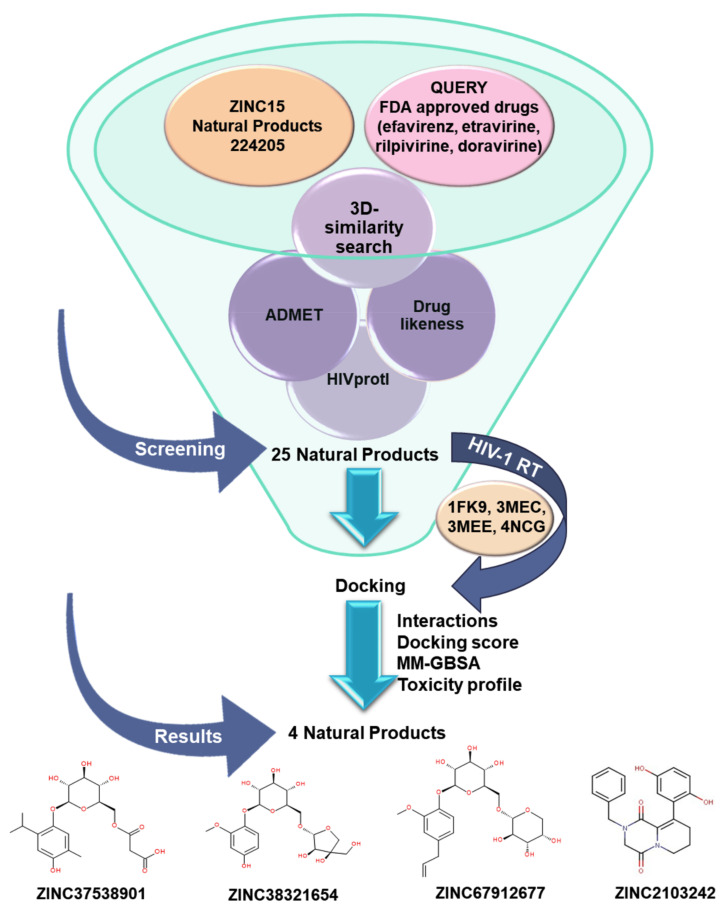
Workflow scheme.

The workflow diagram (Figure 2) followed in this paper involves (1) ZINC15 NPs subset overview, (2) 3D-similarity search involving four FDA approved drugs and 224,205 NPs, (3) ADMETox, (4) HIV protein-inhibitor prediction (HIVprotI) profiles, (5) molecular docking, (6) MM-GBSA simulations, and (7) outcomes analysis.

### 3.2. ZINC15 NPs Subset Analysis

People living with HIV/AIDS are very often using traditional herbal medicines as complementary medicine to improve their immune function, treat symptoms, and minimize side effects of approved medications. The use of natural resources is advantageous because many plant-based bioactive molecules are already found in daily diets. They are less toxic and easy to isolate from plants [48]. Due to these benefits, many plant derivatives have been evaluated or are under study for their possible anti-HIV activity [49,50]. The Lipinski Ro5 rule pretends that for a compound to be assessed as a drug-like molecule, it must not violate more than one criterion: molecular weight (MW) < 500Da, hydrogen bond donor (HBD) ≤ 5, hydrogen bond acceptor (HBA) ≤ 10, and octanol-water partition coefficient(logP) < 5. In this regard, the NP physicochemical properties have been reviewed and observed that many NPs are outside of Lipinski Ro5 space. The 224,205 ZINC15 NPs physicochemical properties acquired using FILTER (OpenEye Scientific Software, Santa Fe, NM, USA) [51] indicated (i) 48,927 compounds with MW ≥ 500, (ii) 4356 compounds with HBA > 10, (iii) 14,980 compounds with HBD > 5, (iv) 28,076 compounds with logP > 5, (v) 38,250 compounds with one Lipinski violation, (vi) 20,784 compounds with two Lipinski violations, (vii) 8764 compounds with three Lipinski violations, and (viii) 783 compounds with four Lipinski violations (Figure S1). To preserve potentially valuable chemical information for anti-HIV activity, all ZINC NP were retained. As an example, cyclosporin A has been approved for a variety of conditions although it violates three out of four Lipinski rules (MW = 1203, logP = 7.5, HBA = 12) [52,53].

### 3.3. Shape-Based Virtual Screening Analysis

**Figure 3:**
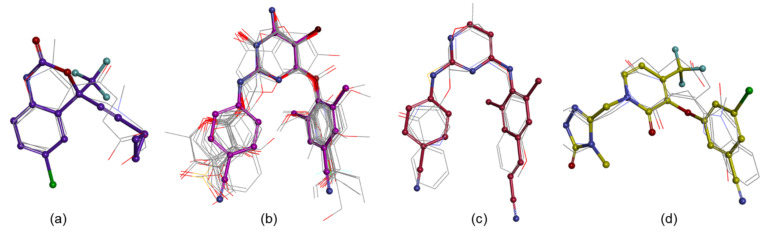
The molecules with ROCS coefficients of TC > 1, ShT > 0.8, and CS > 1.2 overlaid on efavirenz (**a**), etravirine (**b**), rilpivirine (**c**), and doravirine (**d**); the RX ligands are drawn into ball and stick and the selected NPs in line.

The 3D similarity search methods are based on the supposition that molecules with similar structures may have similar activity [54]. The ROCS algorithm [29,30,55,56] searches similar compounds with a query molecule and recovers them based on the molecular shape. The coefficients based on shape, ShapeTanimoto (ShT), with values greater than 0.8 [57], alongside coefficients based on the combination of shape and pharmacophore similarity, ComboScore (CS), with values greater than 1.2 [58], were considered. Also, the TanimotoCombo (TC) coefficients with values greater than 1 were preserved. This coefficient displays how well the volumes of two aligned molecules overlap [55,56]. The ROCS similarity analysis between the templates and the screened molecules suggested that (i) 500 NPs shows TC > 1, 0 NPs shows ShT > 0.8 (however, the first two compounds with ST values close to 0.8 were kept, Table S1), and 493 NPs shows CS > 1.2 towards efavirenz; (ii) 265 NPs shows TC > 1, 31 NPs shows ShT > 0.8, and 158 NPs shows CS > 1.2 towards etravirine; (iii) 70 NPs shows TC > 1, 8 NPs shows ShT > 0.8, and 59 NPs shows CS > 1.2 towards rilpivirine; and (iv) 204 NPs shows TC > 1, 7 NPs shows ShT > 0.8, and 74 NPs shows CS > 1.2 towards doravirine (Figure S2). Of these, two NPs related to efavirenz and doravirine, eighteen NPs related to etravirine, and three NPs related to rilpivirine obeyed all three criteria of TC > 1, ShT > 0.8, and CS > 1.2 (Tables S1 and S2, Figure 3).

### 3.4. ADMETox Analysis

To assess the safety and efficacy of a drug, the pharmacokinetic and pharmacodynamic profiles are essential for the drug development process. Over time, it has been shown that ADMETox drug properties can be a major problem limiting their use. In this context, except eight NPs which violate more than one Lipinski rule, all other NPs fall within the recommended range for MW, logP, HBA, and HBD features (Table S3). The distribution parameters analysis (Table S5) revealed that all 19 out of 25 NPs are not readily able to cross the blood-brain barrier (BBB) and 16 out of 25 NPs to penetrate the central nervous system (CNS). These observations are also supported by the lipophilicity parameter values (Table S3). The absorption parameters denote that the compounds could be absorbed from the intestinal tract upon oral administration. Ten out of 25 selected NPs have low GI absorption (Table S4), indicating a decrease in permeability. Excepting six NPs that showed high Caco-2 cell permeability, 19 NPs did not comply with threshold values. The parameters related to metabolism (Table S6) suggest that, excepting ZINC96113204, ZINC77265897, ZINC96269030, ZINC96113160, ZINC976902, and ZINC2103242, the selected NPs do not influence or inhibit the enzymes of cytochrome P450 (CYP). Thus, it is expected that the latter will not be metabolized in the human body. CYP1A2, CYP2C19, CYP2C9, CYP2D6, CYP2E1, and CYP3A4 play key roles in drug metabolism, of which CYP3A4 is responsible for metabolizing ∼50% of all drugs by itself. It was also shown that CYP2C9 has the ability to metabolize some marketed drugs [59]. Of six compounds that show possible influence or inhibition of CYPs, ZINC96269030, ZINC976902, and ZINC2103242 potentially inhibit the key cytochrome, CYP3A4.

The positive AMES (the bacterial reverse mutation test) toxicity profile (Table S7) indicates that ZINC4340567 and ZINC976902 are potentially mutagenic and therefore may act as a carcinogen because HIV-1 is often linked to mutation. ZINC95486141, ZINC976902, ZINC95911489, ZINC40879757, and doravirine showed values greater than the maximum human-tolerated dose (0.477 log mg/kg/day), indicating a possible toxic behaviour. None of the NPs are considered a likely inhibitor of hERGI, while ZINC95486141, ZINC976902, ZINC77265897, ZINC70455365, ZINC96113204, ZINC96113160, ZINC2103242, etravirine, and rilpivirine are considered possible hERGII inhibitors. The hepatotoxicity parameter shows that six NPs (ZINC976902, ZINC77265897, ZINC96113204, ZINC96113160, and ZINC96269030) and all four FDA-approved drugs could be related to at least one physiological or pathological event, which could be associated with disruption of normal liver function. None of the NPs showed toxicity against T. pyriformis, while ZINC976902 may be associated with a possible toxic Minnow behaviour. The NPs-predicted toxicities (Figure S3) specified that they maintain a relatively lower acute toxicity risk compared to reference drugs.

### 3.5. Antiviral Activity Prediction Analysis

Concerning the antiviral activity evaluated by HIVprotI [37], all 25 ZINC NPs were predicted to show activity against HIV-1 RT with an IC50 range of 2–99.86µM and 27.65–52.46% inhibition (Table S8). Two out of 25 ZINC NPs, ZINC2103242 (52.47%), and ZINC40879757 (50.58%) showed inhibition percentage superior to that of reference drugs, doravirine (45.47%), and etravirine (50.51%), respectively. Due to the inferior ADMETox profile obtained for the ROCS-selected ligands compared to that of rilpirivine, the ligands have not been further evaluated in terms of docking studies. Therefore, 13 out of 25 NPs (bold type in Table S7) predicted with low toxicity and anti-HIV RT activity (Table S8) were subject to docking simulation in 1FK9, 3MEC, and 4NCG receptor active sites.

### 3.6. Docking Analysis

Molecular docking is of significant importance for the new medicine design by accurately predicting the experimental interaction mode and ligands affinity within the appropriate target active site [60]. To study the interactions and conformations of selected NP within our targets, glide SP docking and MM-GBSA studies were carried out.

The docking procedure was validated by computing the RMSD between the X-ray ligands structures and their best docked poses into HIV-1 RT receptors active sites (1FK9, 3MEC, and 4NCG), resulting in RMSD values of 0.548 (for efavirenz), 0.695 (for etravirine), and 0.505 (for doravirine), respectively (Figure S4). After acknowledging the docking protocol quality, the shape-based prioritized NPs were docked into HIV-1 RT. For each NPs, the ligand binding abilities at the receptor active site were evaluated by addressing the following issues: (i) the best pose selection considering an in-depth visual analysis of all the generated poses compared to that of appropriate query, the SP gscore, and the essential interactions with key active site residues (Leu100, Lys101, Lys103, Lys104, Val106, Thr107, Val108, Pro225, Pro236, Val179, Tyr181, Tyr188, Val189, Gly190, Phe227, Trp229, Leu234, His235, and Tyr318 of p66 subunit and Glu138 of p51 subunit) and (ii) the docking analysis validation by computing MM-GBSA binding free energy.

**Figure 4:**
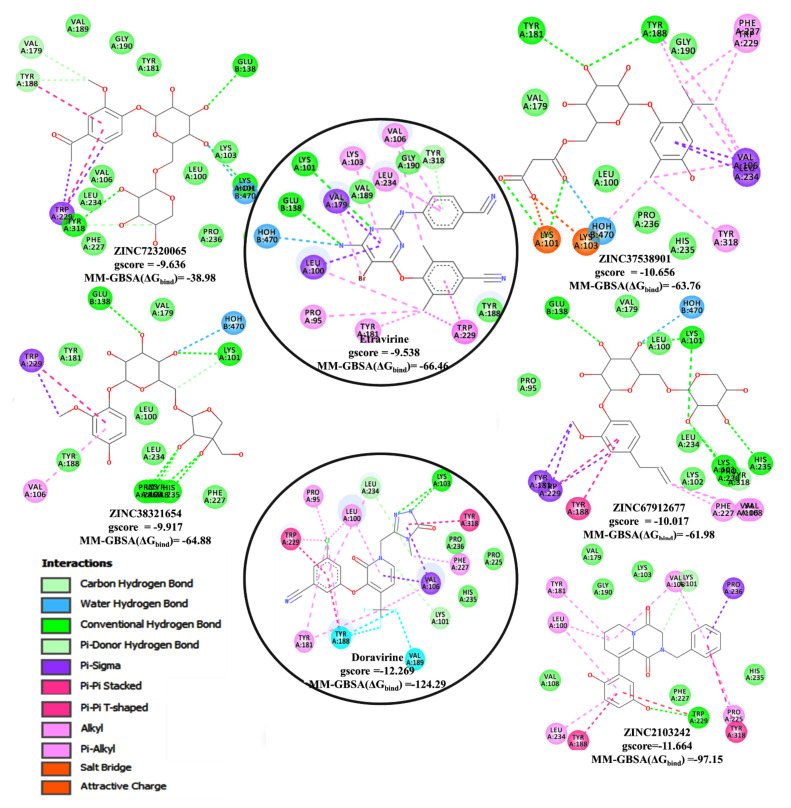
The top docked NPs in the 3MEC/4NCG active sites showing Glide score better than etravirine/doravirine (2D version).

The docking analysis results demonstrated that four out of eleven NPs, ZINC37538901, ZINC67912677, ZINC38321654, and ZINC72320065, have the most favourable docking gscore between −10.656 to −9.636 compared to -9.538 of etravirine (Figure 4). The gscores obtained for the NP-1FK9 (Figure S5) and NP-4NCG (Figure S6) complexes indicated that (ii) two ligands, ZINC2103242 and ZINC514290392, have comparable docking scores of −11.664 and −10.064, respectively, with the reference drug, doravirine (−12.269) and (ii) none of the remaining NP ligands have higher scores than efavirenz. These scores suggest that the newly predicted molecules might have better inhibitory activity against HIV-RT. In this condition, only the NPs-receptor complexes, NP-3MEC, and NP-4NCG (Figure 4, Figures S6 and S7) were further discussed. Both NP complexes were visually inspected by comparing the atomic coordinates and the interactions of each docked NP with that of the reference drugs by employing Discovery Studio Visualizer facilities. Analysis of the binding position occupied by NPs in the docked complexes indicated that all ligands are located within the HIV-1 RT receptor binding cavity. Furthermore, all prioritized poses of NPs within the appropriate receptor were analyzed for the interaction profile involving hydrogen bonding (H-bond), π–π stacked, π-π T-shaped, π–sigma, π-alkyl, attractive charge/salt bridge, and water H-bonds (Figure 4, Figures S5–S7 and Table S9) and the minimum interaction energy estimated using glide gscore (Figure 4 and Figures S5–S7).

Generally, the conventional hydrogen bonds (H-bond) with Lys101, Glu138, and Tyr318 were observed for all NPs-3MEC complexes (Figure 4, Figure S7) except ZINC37538901, which made H-bonds with Lys101, Tyr181, and Tyr188. Additional carbon hydrogen bonds formed by hydroxyl groups attached to oxan and oxolan rings with His235, Lys103, Tyr318, and Pro236 were noticed for ZINC67912677, ZINC38321654, and ZINC72320065. All four NPs connect to the receptor by forming a large number of hydrophobic interactions, such as (i) π-alkyl (Val106, Leu234, Lys103, Trp229, Tyr181, Tyr188, Phe227, Tyr318), (ii) π-σ (Trp229, Val179, Leu100, Tyr188, Val106, Leu234), (iii) π-π stacked (Tyr188), (iv) π-π T-shaped (Trp229), (v) attractive charge/salt bridge (Lys101, Lys103), and (vi) water H-bonds (HOH470). The presence of oxan and oxolan rings with hydroxyl substituents in ligands structures favors a large number of H-bond interactions.

As observed from Figure 4 and Figure S6, ZINC2103242 and ZINC514290392 adopted similar binding patterns with the reference drug, doravirine, by establishing H-bonds with Trp229 and Lys101. Also, both ligands bridge to the receptor, forming hydrophobic interactions, such as (i) π-alkyl (Val106, Pro225, Leu234, Lys100, Trp229, Tyr181), (ii) π-π stacked (Tyr188, Tyr318), (iii) π-σ (Val106, Pro236), (iv) carbon hydrogen bonds (Lys101), (v) π-π T-shaped (Tyr181,Trp229), and (vi) attractive charge/salt bridge (Tyr318, Lys103). More detailed information on the established interaction of the ligands with the active site residues and their lengths are provided in Table S9. The H-bonds together with the additional hydrophobic and electrostatic interactions anchor ligands within the active site and implicitly stabilize their biding orientation mode. Moreover, the presence of π-π stacked H-bonds and hydrophobic interactions strengthen their crucial importance for allosteric NNRTIs binding and the possibility of not developing resistance to RT mutation. It is well known that the loss of π-π stacked interactions may have unfavorable effects on inhibitory activity. Likewise, Tyr188, Tyr181, Lys103, and Trp229 residues had a substantial contribution to the interaction energies of the systems. Our docking outcomes are in line with previous reports [61,62].

### 3.7. MM-GBSA Binding Free Energy Analysis

**Figure 5:**
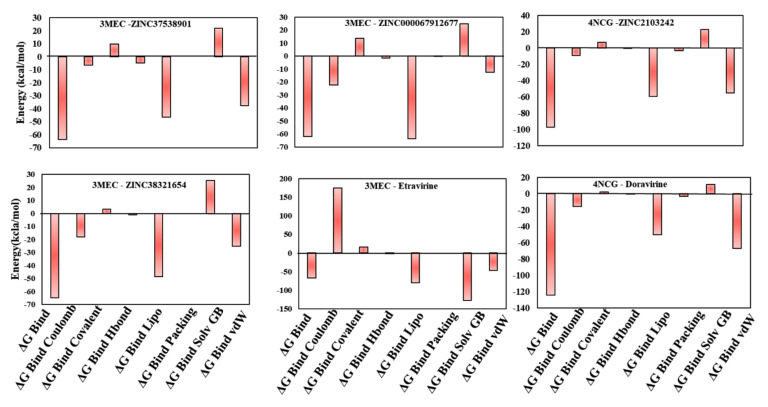
Contributions of different energy components to the total free binding energies of the ligand-receptor complexes (kcal mol^−1^).

The docking results indicated that six screened NPs might have the ability to inhibit HIV-1 RT. Their protein-ligand affinities were evaluated by computing MM-GBSA binding free energies. Prime MM-GBSA (ΔG_bind_) range was from −64.88 kcal/mol (3MEC-ZINC38321654), −63.76 kcal/mol (3MEC-ZINC37538901), and −61.98 kcal/mol (3MEC-ZINC67912677) to −38.98 kcal/mol (3MEC-ZINC72320065). As observed, all compounds have comparable free binding energies with that of etravirine (MM-GBSA ΔG_bind_ of −66.46 kcal/mol) except for ZINC72320065 (Figure 4, Figure 5, Figure S7). HIV-1 RT complexes 4NCG-ZINC2103242 presented an acceptable MM-GBSA ΔG_bind_ of −97.15 kcal/mol compared to that of doravirine, −124.29 kcal/mol, while for 4NCG-ZINC514290392, it was much lower (Figure 4, Figure 5, Figure S6). Moreover, analysis of dissociation energy components highlighted the major contribution of nonpolar solvation (ΔG_bind_ Lipo) and the van der Waals interactions (ΔG_bind_ vdW) parameters in the stability of docked complexes and ligands biding affinities (Figure 5). It was also observed that both ΔG_bind_ covalent and ΔG_bind_ solv GB components delivered unfavorable energies for ligands binding.

Based on all the investigations, it was noticed that ZINC37538901, ZINC38321654, ZINC67912677, and ZINC2103242 display small RMSDs, a significant number of key interactions with active site residues, high docking scores, favorable ADMETox and HIVprotI profiles, and comparable binding free energies with reference drugs. Consequently, we propose these four NPs to be investigated as potential nontoxic inhibitors for HIV1-RT, with ZINC2103242 showing to be the most promising candidate among all four.

### 3.8. Known Therapeutic Benefits of the Proposed Natural Products

ZINC37538901 is a natural derivative containing phenyl-β-d-glucopyranoside core known to develop anticancer, anti-inflammatory, antiseptic, and many other activities [63].

Canthoside D (ZINC38321654), a phenolic compound isolated from arial parts of Salsola tetragona specie, is known to possess anticancer, antimicrobial, anti-inflammatory, antioxidant, antidepressant, and antihypertensive activities [64].

Geoside (ZINC67912677), one out of more than 30 steviol glycosides, is a natural sweetness compound extracted from Stevia rebaudiana leaves. The steviol glycosides are used in the food industry, especially as sweeteners in fruit juices. They also exhibit anti-inflammatory, antibacterial, antiviral, antitumor, antihyperglycemic, antioxidant activities, etc. In short, stevia has zero calories and many benefits. Like most natural compounds, they are safe for human health and could be consumed without restriction by diabetics [65].

ZINC2103242 contains a pyridopyrazines core that is known to manifest antibacterial, antimalarial, antitumoral, antiallergics, and antidepressant activities together with diuretic, virucid, anxiolitic, hypnnotic, and analgesic effects. Also, the pyridopyrazines analogs were employed as modulators of signal transduction pathways to treat various physiological and/or pathophysiological conditions [66].

The beneficial effects of natural products and of their corresponding plant-sources argue that they could be used as safe, preventive chemotherapeutic agents as well as a viable solution to reduce the side effects of conventional medicine.

## 4. Conclusions

3D-similarity search, ADMETox, molecular docking, and MM-GBSA simulations were performed within 224,205 natural compounds of the ZINC15 NPs subset to evaluate potential new antiviral agents against HIV-1 RT. The in silico analysis revealed that four (ZINC37538901, ZINC67912677, ZINC38321654, and ZINC2103242) out of twenty-five selected natural products fulfilled all the parameters investigated, such as 3D-similarity coefficients, ADMETox parameters, the predicted IC50/percent inhibition of HIV RT protein, docking scores, and free binding energies. Moreover, the presence of oxan and oxolan rings with hydroxyl substituents and of 3,6,7,8-tetrahydro-2H-pyrido [1,2-a]pyrazine-1,4-dione core in NP molecules favour the hydrogen bonds’ interaction and implicitly superior docking scores and comparable free energies to that of the query compounds, the FDA-approved drugs, etravirine and doravirine. The docking outcomes suggested that residues Lys101, Tyr181, Tyr188, Trp229, and Tyr318, involved in essential hydrogen bonding and п-п stacked interaction which stabilized ZINC NPs in the HIV-1 RT active site, played essential roles for anti-HIV activity. Out of four candidates, ZINC2103242 proved to be the most promising in terms of drug metabolism and safety profile. Despite the outstanding use of computational techniques in the process of developing effective therapies, there are some limitations and, at the same time, challenges to implement these studies in such a way as to simulate the behavior of living organisms. Therefore, we proposed these four ZINC NPs with nontoxic predicted qualities, with special focus on ZINC2103242, to be further explored as possible HIV-1 RT inhibitors by combining computational outcomes with the experimental and clinical investigation to enrich the rate of drug discovery success.

## Data Availability

The data that support the results and findings of this study are available from the corresponding author upon request.

## Supplementary Materials

The following are available at https://www.mdpi.com/article/10.3390/life11070722/s1, Figure S1: Distribution of drug-like properties for ZINC15 NPs subset; Figure S2: The first ten aligned molecules with ROCS: Efavirenz-TanimotoCombo values criteria (a), ShapeTanimoto values (b), TanimotoCombo values (c), Etravirine-TanimotoCombo values criteria (d), ShapeTanimoto values (e), TanimotoCombo values (f), Rilpirivine-TanimotoCombo values criteria (g), ShapeTanimoto values (h), TanimotoCombo values (i), Doravirine-TanimotoCombo values criteria (j), ShapeTanimoto values (k), TanimotoCombo values (l); Table S1: The best NPs 3D-similarity coefficients vs. efavirenz (EFV), etravirine (ETV), rilpivirine (RPV), and doravirine (DOR). 2D structures of queries molecules and selected NPs (TC (Tanimoto Combo) >1, ShT (Shape Tanimoto) >0.8, and CS (Combo Score) >1.2); Table S2: Unique BMFs of queries molecules and selected NPs; Table S3: Physicochemical properties, lipophilicity, drug-likeness, and medicinal chemistry parameters# for selected NPs and drugs calculated with Swiss ADME and pkCSM*; Table S4: Absorption parameters for selected NPs and drugs calculated with pkCSM; Table S5: Distribution and excretion parameters for selected NPs and drugs calculated with pkCSM; Table S6: Metabolism parameters for selected NPs and drugs calculated with pkCSM; Table S7: Toxicity parameters for selected NPs and drugs calculated with pkCSM; Figure S3: Pie chart representation of the alerts distribution for selected NPs; Table S8: Approved drugs, ZINC NPs, and their predicted antiviral activity (IC50 and procent inhibition) against HIV-1 RT using HIVprotI online platform (bioinfo.imtech.res.in/manojk/hivproti/, accessed on 8 June 2021); Figure S4: Efavirenz (RX-purple; the docked pose—grey) (a), Etravirine (RX-magenta; the docked pose—grey) (b), and Doravirine (RX-yelow; the docked pose—grey) (c); Figure S5: The selected docked NP (non-toxic) in the 1FK9 binding site— efavrinez and ZINC771797 (2D presentation). In the circle, the efavrinez (purple) and the selected NPs in 1FK9 are presented (3D variant). The green color indicates the hydrogen bonding area; Figure S6: The selected docked NP in the 4NCG binding site—doravirine and ZINC514290392 (2D presentation). In the circle, the doravirine (yelow) and the selected NPs in 4NCG are presented (3D variant). The green color indicates the hydrogen bonding area; Figure S7: The selected docked NP (non-toxic) in the 3MEC binding site (2D presentation). In the circle, the etravirine (magenta) and the selected NPs in 3MEC are presented (3D variant). The green color indicates the hydrogen bonding area. MM-GBSA(ΔGbind)is expressed in kcal/mol; Table S9: Docked interaction analysis of selected NPs with target proteins 3MEC and 4NCG.
