# Supplementary material for: Small Molecules of Natural Origin as Potential Anti-HIV Agents: A Computational Approach
Source: Life (Basel). 2021 Jul 20;11(7):722. doi: 10.3390/life11070722 (PMC8303883; doi:10.3390/life11070722)
Supplement: Supplementary file 1 [file life-11-00722-s001.zip › life-1273268-supplementary.pdf]

Supplemenatry Materials

Small molecules of natural origin as potential Anti-HIV agents: a computational approach

Luminita Crisan [1],* and AlinaBora [1],*

[1] "Coriolan Dragulescu" Institute of Chemistry, 24 M. Viteazu Ave, 300223 Timisoara, Romania; lumi_crisan@acad-icht.tm.edu.ro (L.C.); alina_bora@acad-icht.tm.edu.ro (A.B.)

* Correspondence: alina_bora@acad-icht.tm.edu.ro ; lumi_crisan@acad-icht.tm.edu.ro

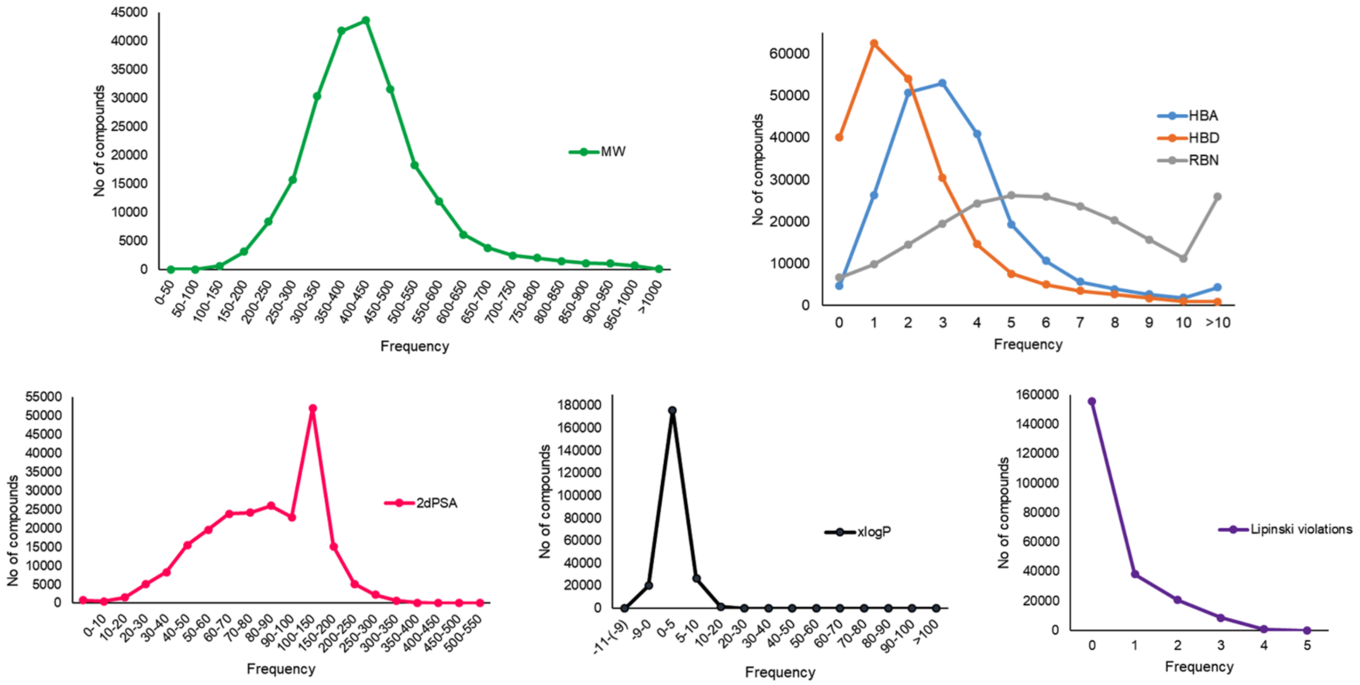

Figure S1. Distribution of drug-like properties for ZINC15 NPs subset.

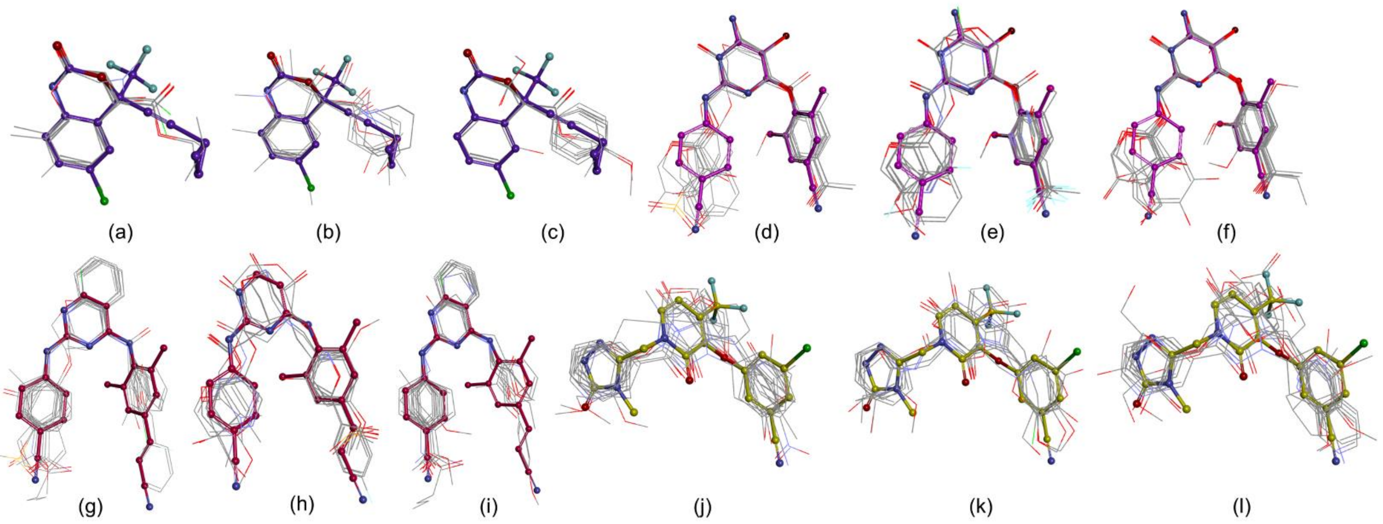

Figure S2. The first ten aligned molecules with ROCS: Efavirenz - TanimotoCombo values criteria (a), ShapeTanimoto values (b), TanimotoCombo values (c); Etravirine - TanimotoCombo values criteria (d), ShapeTanimoto values (e), TanimotoCombo values (f); Rilpirivine - TanimotoCombo values criteria (g), ShapeTanimoto values (h), TanimotoCombo values (i); Doravirine - TanimotoCombo values criteria (j), ShapeTanimoto values (k), TanimotoCombo values (l)

Table S1. The best NPs 3D-similarity coefficients vs efavirenz (EFV), etravirine (ETV), rilpivirine (RPV), and doravirine (DOR); 2D structures of queries molecules and selected NPs (TC (Tanimoto Combo) >1, ShT (Shape Tanimoto) >0.8 and CS (Combo Score) >1.2)

Name	2D-Structures	Name	2D-Structures
Etravirine	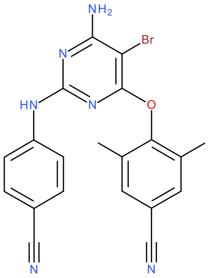	Rilpivirine	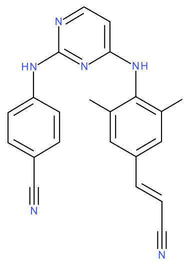
	BMF-1		BMF-1
Efavirenz	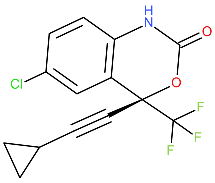	Doravirine	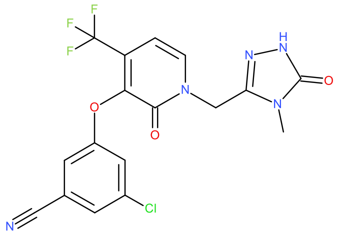
	BMF-2		BMF-3
ZINC142491991<br>Query – ETV<br>TC = 1.154<br>ShT = 0.844<br>CS = 1.403	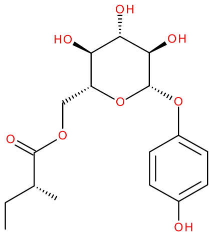	ZINC142492210<br>Query – ETV<br>TC = 1.13<br>ShT = 0.825<br>CS = 1.384	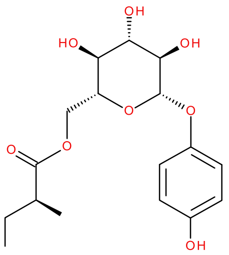
	BMF-4		BMF-4
ZINC37538901<br>Query – ETV<br>TC = 1.089<br>ShT = 0.800<br>CS = 1.390	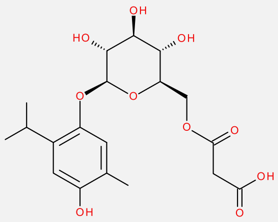	ZINC95911489<br>Query – ETV<br>TC = 1.076<br>ShT = 0.805<br>TC = 1.348	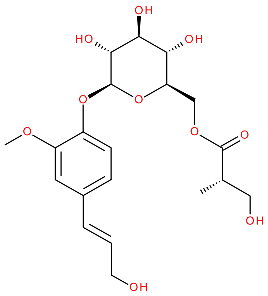
	BMF-4		BMF-4
ZINC33975071<br>Query – ETV<br>TC = 1.147<br>ShT = 0.834<br>CS = 1.528	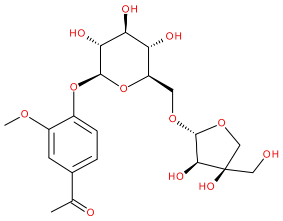	ZINC96023826<br>Query – ETV<br>TC = 1.104<br>ShT = 0.811<br>CS = 1.450	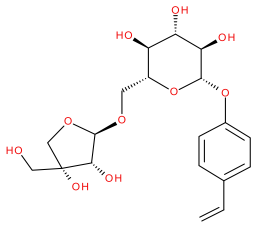

BMF-5

BMF-5

ZINC38321654
Query – ETV
TC = 1.096
ShT = 0.813
CS = 1.475

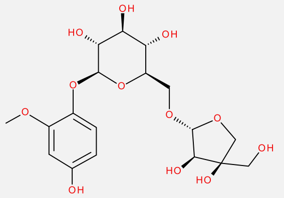

ZINC38321675
Query – ETV
TC = 1.077
ShT = 0.809
CS = 1.404

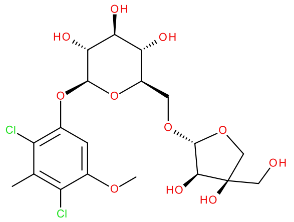

BMF-5

BMF-5

ZINC238760037
Query – ETV
TC = 1.116
ShT = 0.806
CS = 1.495

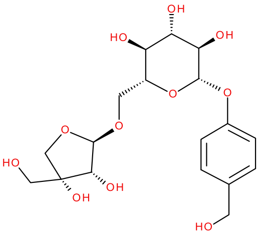

ZINC70455365
Query – ETV
TC = 1.104
ShT = 0.808
CS = 1.492

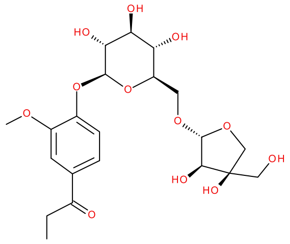

BMF-5

BMF-5

ZINC96113204
Query – RPV / ETV
TC = 1.061 / 1.086
ShT = 0.801 / 0.814
CS = 1.214 / 1.203

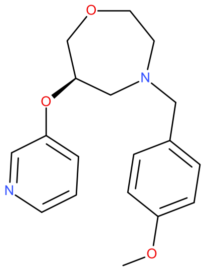

ZINC77265897
Query – RPV
TC = 1.045
ShT = 0.801
CS = 1.215

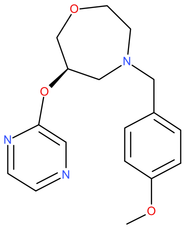

BMF-6

BMF-6

ZINC96269030
Query – ETV
TC = 1.153
ShT = 0.810
CS = 1.274

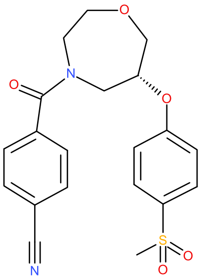

ZINC96113160
Query – ETV
TC = 1.095
ShT = 0.824
CS = 1.211

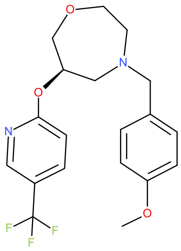

BMF-6

BMF-6

ZINC72320065
Query – ETV
TC = 1.094
ShT = 0.806
CS = 1.457

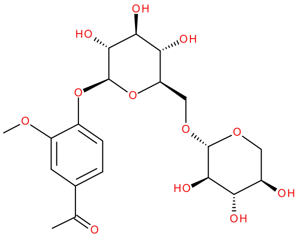

ZINC67912677
Query – ETV
TC = 1.080
ShT = 0.814
CS = 1.425

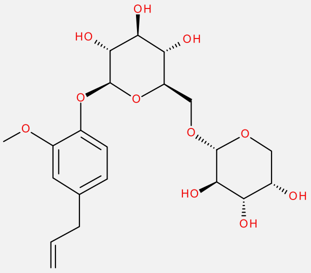

BMF-7

BMF-7

ZINC95911423
Query – ETV
TC = 1.064
ShT = 0.802
CS = 1.406

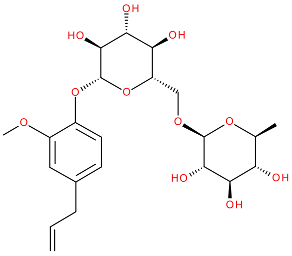

ZINC976902
Query – RPV
TC = 1.074
ShT = 0.801
TC = 1.209

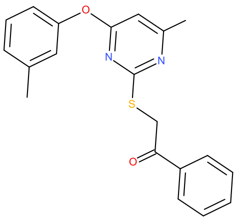

BMF-7

BMF-8

ZINC40879757
Query – ETV
TC = 1.118
ShT = 0.823
CS = 1.383

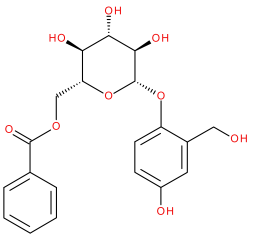

BMF-9

ZINC95486141
Query – ETV
TC = 1.011
ShT = 0.813
CS = 1.293

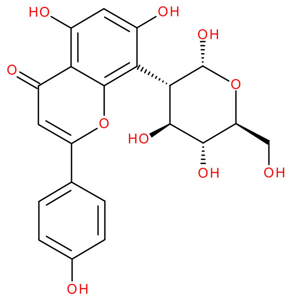

BMF-10

ZINC2103242
Query – DOR
TC = 1.078
ShT = 0.839
TC = 1.247

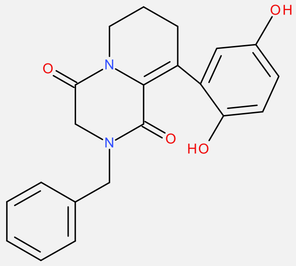

BMF-11

ZINC514290392
Query – DOR
TC = 1.035
ShT = 0.813
TC = 1.277

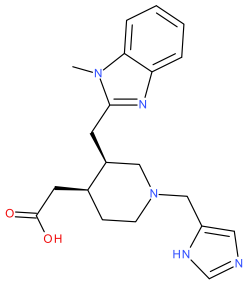

BMF-12

ZINC4340567
Query – EFV
TC = 1.163
ShT = 0.790
CS = 1.537

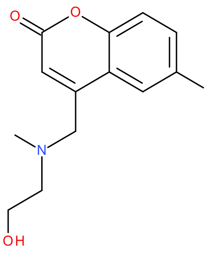

BMF-13

ZINC771797
Query – EFV
TC = 1.141
ShT = 0.789
CS = 1.506

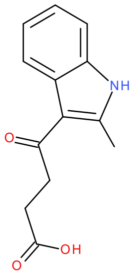

BMF-14

The fourteen distinct scaffolds of twenty selected NPs, as illustrated by BMFs (Table S2), were generated using the InstantJChem tool [1]. The BMF enables to group structures based on the included frameworks such as individual or condensed rings and atoms that connect them, without substituents. A significant rate of scaffolds (Tables S1 and S2) were observed for all predicted NPs even if they were selected using the same template. Again, we emphasize the scaffold hopping abilities of 3D-similarity search with ROCS, which is capable to provide significant structural heterogeneity [2,3].

References:
1. InstantJchem 20.15.0, 2020, ChemAxon, http://www.chemaxon.com
2. Pacureanu, L.; Avram, S.; Bora, A.; Kurunczi, L.; Crisan L. Portraying the selectivity of GSK-3 inhibitors towards CDK-2 by 3D similarity and molecular docking. *Struct. Chem.* **2019**, *30*, 911–923. doi.org/10.1007/s11224-018-1224-z.
3. ROCS v. 3.2.1.4, OpenEye Scientific Software, Santa Fe, NM., www.eyesopen.com

Table S2. Unique BMFs of queries molecules and selected NPs

Name	2D-Structures	Name	2D-Structures
BMF-1	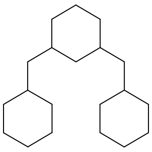	BMF-2	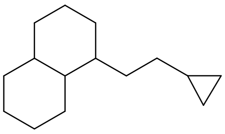
BMF-3	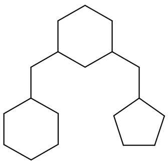	BMF-4	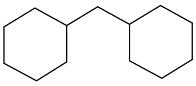
BMF-5	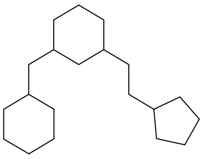	BMF-6	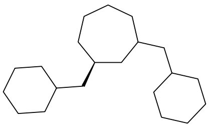
BMF-7	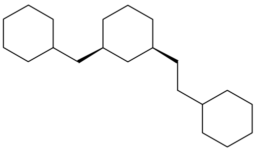	BMF-8	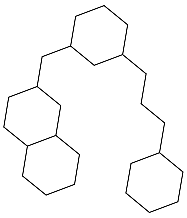
BMF-9	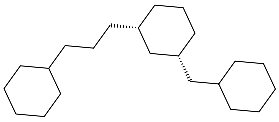	BMF-10	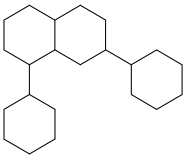
BMF-11	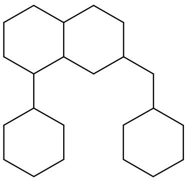	BMF-12	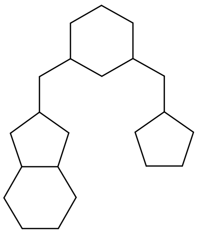
BMF-13	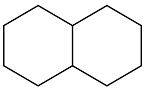	BMF-14	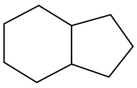

Table S3. Physicochemical properties, lipophilicity, druglikeness and medicinal chemistry parameters[#] for selected NPs and drugs calculated with Swiss ADME and pkCSM[*]

Molecule	Swiss ADME											pkCSM	
	MW	RBN	HBA	HBD	TPSA	iLOGP	XLOGP3	WLOGP	MLOGP	Lipinski #violations	PAINS #alerts	LOGP	PSA
ZINC4340567	247.29	18	4	4	53.68	2.41	0.98	1.37	1.33	0	0	1.526	105.458
ZINC771797	231.25	17	4	3	70.16	1.37	1.56	2.52	1.03	0	0	2.524	98.264
ZINC142491991	356.37	7	8	4	125.68	1.63	0.07	0.17	-0.27	0	0	0.168	145.119
ZINC33975071	460.43	8	12	6	184.60	2.76	-2.36	-2.46	-3.04	2	0	-2.459	183.024

ZINC40879757	406.38	7	9	5	145.91	1.88	0.11	-0.22	-0.33	0	0	-0.072	165.876
ZINC96023826	414.4	7	10	6	158.30	1.83	-1.24	-2.14	-2.02	1	0	-2.027	166.694
ZINC38321654	434.39	7	12	7	187.76	1.41	-2.4	-2.96	-3.14	2	0	-2.956	170.926
ZINC72320065	460.43	7	12	6	184.60	2.75	-2.66	-2.46	-3.04	2	0	-2.460	183.024
ZINC96113204	314.38	5	5	0	43.82	2.98	2.02	1.84	0.98	0	0	2.370	136.357
ZINC38321675	501.31	7	11	6	167.53	2.28	-0.42	-1.05	-1.44	3	0	-1.046	193.104
ZINC95911423	472.48	8	11	6	167.53	2.69	-0.84	-1.55	-1.85	2	0	-1.546	190.902
ZINC77265897	315.37	5	6	0	56.71	2.68	1.28	1.23	0.34	0	0	1.765	135.577
ZINC96269030	400.45	5	6	0	105.08	2.32	1.70	2.58	1.01	0	0	1.882	163.569
ZINC142492210	356.37	7	8	4	125.68	2.75	0.07	0.17	-0.27	0	0	0.168	145.119
ZINC238760037	418.39	7	11	7	178.53	1.57	-2.90	-3.33	-2.92	2	0	-3.178	165.813
ZINC70455365	474.46	9	12	6	184.60	2.26	-1.89	-2.07	-2.82	2	0	-2.069	189.389
ZINC96113160	382.38	6	8	0	43.82	2.79	3.24	4.01	2.23	0	0	3.389	155.219
ZINC37538901	414.4	8	10	5	162.98	1.74	0.43	0.03	-0.62	0	0	0.028	166.805
ZINC67912677	458.46	8	11	6	167.53	2.25	-1.27	-1.93	-2.06	2	0	-1.934	184.537
ZINC95911489	428.43	10	10	5	155.14	2.14	-0.66	-1.05	-1.21	0	0	-0.941	173.432
ZINC95486141	432.38	3	10	7	181.05	1.59	0.52	0.09	-1.61	1	0	0.092	173.994
ZINC976902	386.47	6	4	0	77.38	3.62	5.97	5.71	3.52	0	0	5.706	167.16
ZINC2103242	364.39	27	3	4	81.08	2.53	1.98	1.56	1.43	0	0	2.474	156.503
ZINC514290392	367.44	27	6	5	87.04	1.71	-0.7	1.92	1.26	0	0	2.452	157.597
Efavirenz	315.67	1	5	1	38.33	2.68	4.01	4.67	3.61	0	0	4.073	122.963
Etravirine	435.28	4	5	2	120.64	3.22	4.45	4.73	2.48	0	0	4.717	170.101
Rilpivirine	366.42	5	4	2	97.42	3.23	4.55	4.88	2.37	0	0	4.989	163.370
Doravirine	425.75	5	8	1	105.7	2.64	2.09	3.81	1.89	0	0	2.655	164.891

#Molecular weight (MW) < 500 Dalton; hydrogen bond donor (HBD) ≤ 5; hydrogen bond acceptor (HBA) ≤ 10; polar surface area (PSA)>130Å^2 - the cell membrane permeability is limited.; the octanol–water partition coefficient (logP) < 5

Table S4. **Absorption** parameters for selected NPs and drugs calculated with pkCSM

Molecule	Water solubility (log mol/L)	Caco2 permeability (log cm/s) high Caco2 permeability >0.9	Human intestinal absorption (% Absorbed) poorly absorbed <30%	Skin Permeability low skin permeability, log Kp>-2.5	P-glycoprotein substrate	P-glycoprotein I inhibitor	P-glycoprotein II inhibitor
ZINC4340567	-1.399	1.215	93.462	-2.885	Yes	No	No
ZINC771797	-2.77	0.869	91.061	-2.647	No	No	No
ZINC142491991	-1.761	0.429	57.700	-2.737	Yes	No	No
ZINC33975071	-1.403	-0.708	28.696	-2.735	Yes	No	No
ZINC40879757	-3.437	0.344	54.471	-2.737	Yes	No	No
ZINC96023826	-1.248	-0.721	29.314	-2.737	No	No	No
ZINC38321654	-2.077	-0.602	21.989	-2.735	Yes	No	No
ZINC72320065	-1.407	-0.697	28.764	-2.735	Yes	No	No
ZINC96113204	-2.509	1.261	94.231	-2.786	No	No	No
ZINC38321675	-2.696	-0.192	27.117	-2.735	Yes	No	No
ZINC95911423	-1.611	-0.806	31.733	-2.736	Yes	No	No
ZINC77265897	-2.465	1.242	94.560	-2.828	No	No	No
ZINC96269030	-3.936	1.193	77.547	-2.962	No	Yes	Yes
ZINC142492210	-1.761	0.429	57.700	-2.737	Yes	No	No
ZINC238760037	-1.187	-0.802	20.038	-2.737	No	No	No
ZINC7455365	-1.593	-0.721	29.591	-2.735	Yes	No	No
ZINC96113160	-3.307	1.126	90.763	-2.904	No	Yes	Yes
ZINC37538901	-2.581	0.070	30.157	-2.736	Yes	No	No
ZINC67912677	-1.535	-0.798	30.908	-2.736	Yes	No	No
ZINC95911489	-2.23	0.064	43.156	-2.748	Yes	No	No
ZINC95486141	-3.416	0.471	49.860	-2.735	Yes	No	No
ZINC976902	-5.778	1.070	95.217	-2.731	No	Yes	Yes
ZINC2103242	-3.686	0.888	89.213	-2.981	Yes	Yes	Yes
ZINC514290392	-2.892	0.895	86.189	-2.735	Yes	No	No
Efavirenz	-5.606	1.516	89.984	-3.09	No	Yes	No

Etravirine	-3.908	0.285	86.399	-2.753	Yes	Yes	Yes
Rilpivirine	-4.245	0.486	93.213	-2.772	Yes	Yes	Yes
Doravirine	-3.225	0.364	82.757	-2.741	Yes	No	No

Table S5. Distribution and Excretion parameters for selected NPs and drugs calculated with pkCSM

Molecule	Human volume of distribution (VDss) (log L/kg) VDss low, logVDss<-0.15 VDss high, logVDss>0.45	Human fraction unbound (Fu)	BBB permeability (log BB) readily cross the BBB, logBB>0.3 poorly distributed, logBB<-1	CNS permeability (log PS) to Penetrate the CNS, logPS>-2 unable to penetrate the CNS, logPS<-3	Total Clearance (log ml/min/kg)	Renal OCT2 substrate
ZINC4340567	0.7	0.408	-0.006	-2.511	0.814	No
ZINC771797	-0.495	0.211	-0.101	-2.438	0.627	No
ZINC142491991	0.347	0.540	-1.04	-3.848	1.011	No
ZINC33975071	0.064	0.541	-1.579	-4.741	0.781	No
ZINC40879757	-0.209	0.209	-1.247	-3.97	0.199	No
ZINC96023826	0.119	0.611	-1.279	-4.443	0.917	No
ZINC38321654	0.345	0.541	-1.542	-4.954	0.653	No
ZINC72320065	0.071	0.539	-1.55	-4.694	0.830	No
ZINC96113204	0.494	0.491	0.335	-2.196	0.818	Yes
ZINC38321675	-0.439	0.438	-1.707	-4.876	1.027	No
ZINC95911423	0.024	0.565	-1.433	-4.487	1.012	No
ZINC77265897	0.24	0.501	-0.030	-2.836	0.756	No
ZINC96269030	-0.537	0.027	-0.713	-2.550	0.780	No
ZINC142492210	0.347	0.540	-1.04	-3.848	1.011	No
ZINC238760037	0.062	0.676	-1.39	-4.746	0.909	No
ZINC70455365	0.03	0.499	-1.612	-4.656	0.847	No
ZINC96113160	0.323	0.408	0.214	-2.053	0.595	No
ZINC37538901	-0.444	0.385	-1.438	-4.049	0.806	No
ZINC67912677	0.022	0.577	-1.410	-4.546	0.997	No
ZINC95911489	-0.399	0.543	-1.363	-4.173	0.296	No

ZINC95486141	0.192	0.031	-1.579	-4.459	0.621	No
ZINC976902	-0.091	0.140	0.187	-1.847	0.188	No
ZINC2103242	*0.143*	*0.085*	*-0.696*	*-2.335*	*0.233*	*No*
ZINC514290392	-0.095	0.377	-0.931	-3.508	0.412	Yes
Efavirenz	0.045	0.029	0.036	-1.651	-0.147	No
Etravirine	0.069	0.014	-0.496	-1.863	-0.426	No
Rilpivirine	0.400	0	-0.100	-1.770	0.209	No
Doravirine	*0.135*	*0.137*	*-1.713*	*-2.790*	*-0.102*	*No*

Table S6. Metabolism parameters for selected NPs and drugs calculated with pkCSM

Molecule	CYP2D6 substrate	CYP3A4 substrate	CYP1A2 inhibitor	CYP2C19 inhibitor	CYP2C9 inhibitor	CYP2D6 inhibitor	CYP3A4 inhibitor
ZINC4340567	No	No	Yes	No	No	No	No
ZINC771797	No	Yes	No	No	No	No	No
ZINC142491991	No	No	No	No	No	No	No
ZINC33975071	No	No	No	No	No	No	No
ZINC40879757	No	No	No	No	No	No	No
ZINC96023826	No	No	No	No	No	No	No
ZINC38321654	No	No	No	No	No	No	No
ZINC72320065	No	No	No	No	No	No	No
**ZINC96113204**	**No**	**Yes**	**Yes**	**No**	**No**	**Yes**	**No**
ZINC38321675	No	No	No	No	No	No	No
ZINC95911423	No	No	No	No	No	No	No
**ZINC77265897**	**No**	**Yes**	**Yes**	**No**	**No**	**No**	**No**
**ZINC96269030**	**No**	**Yes**	**No**	**Yes**	**No**	**No**	**Yes**
ZINC142492210	No	No	No	No	No	No	No
ZINC238760037	No	No	No	No	No	No	No
ZINC70455365	No	No	No	No	No	No	No
**ZINC96113160**	**No**	**Yes**	**Yes**	**No**	**No**	**Yes**	**No**

ZINC37538901	No	No	No	No	No	No	No
ZINC67912677	No	No	No	No	No	No	No
ZINC95911489	No	No	No	No	No	No	No
ZINC95486141	No	No	No	No	No	No	No
**ZINC976902**	**No**	**Yes**	**Yes**	**Yes**	**Yes**	**No**	**Yes**
**ZINC2103242**	**No**	**Yes**	**No**	**Yes**	**No**	**No**	**Yes**
ZINC514290392	No	No	No	No	No	No	No
Efavirenz	No	Yes	No	Yes	Yes	No	No
Etravirine	No	Yes	Yes	Yes	Yes	No	Yes
Rilpivirine	No	Yes	Yes	Yes	Yes	No	Yes
Doravirine	No	No	Yes	Yes	No	No	Yes

Table S7. Toxicity parameters for selected NPs and drugs calculated with pkCSM

Molecule	AMES toxicity[a]	Max. Human tolerated Dose[b] (log mg/kg/day) Toxic effect>0.477 log mg/kg/day	hERG I inhibitor[c]	hERG II inhibitor[d]	Oral Rat Acute Toxicity[e] (LD50) (mol/kg)	Oral Rat Chronic Toxicity[f] (LOAEL) (log mg/kg_bw/day)	Hepatotoxicity[g]	Skin Sensitization[h]	T.Pyriformis toxicity[i] (log ug/L)	Minnow toxicity[j] (log mM)
ZINC4340567	Yes	0.175	No	No	2.305	2.372	No	No	0.810	1.711
**ZINC771797**	No	0.424	No	No	2.206	2.22	No	No	0.557	0.545
**ZINC142491991**	No	0.077	No	No	2.149	3.286	No	No	0.285	2.077
**ZINC33975071**	No	0.036	No	No	2.895	3.866	No	No	0.285	7.149
ZINC40879757	No	0.606	No	No	2.410	3.757	No	No	0.285	1.954
**ZINC96023826**	No	0.138	No	No	2.825	3.896	No	No	0.285	5.374
**ZINC38321654**	No	0.22	No	No	2.774	3.808	No	No	0.285	7.359
ZINC72320065	No	0.014	No	No	2.906	3.873	No	No	0.285	7.197
ZINC96113204	No	-0.171	No	Yes	3.212	1.198	Yes	No	0.332	1.398
**ZINC38321675**	No	0.254	No	No	3.432	3.717	No	No	0.285	5.452
**ZINC95911423**	No	-0.028	No	No	2.905	3.818	No	No	0.285	5.951

ZINC77265897	No	0.054	No	Yes	3.197	1.179	Yes	No	0.313	1.904
ZINC96269030	No	0.106	No	No	1.984	1.481	Yes	No	0.795	1.259
**ZINC142492210**	No	0.077	No	No	2.149	3.286	No	No	0.285	2.077
**ZINC238760037**	No	0.255	No	No	2.735	3.933	No	No	0.285	6.071
ZINC70455365	No	-0.082	No	Yes	2.976	3.997	No	No	0.285	6.848
ZINC96113160	No	-0.212	No	Yes	3.452	1.03	Yes	No	0.356	1.307
**ZINC37538901**	No	0.276	No	No	2.273	3.281	No	No	0.285	0.757
**ZINC67912677**	No	0.018	No	No	2.865	3.856	No	No	0.285	6.068
ZINC95911489	No	0.746	No	No	2.214	3.088	No	No	0.285	4.315
ZINC95486141	No	0.93	No	Yes	3.007	3.18	No	No	0.285	3.977
ZINC976902	Yes	0.852	No	Yes	2.663	0.992	Yes	No	0.294	-4.961
**ZINC2103242**	No	-0.558	No	Yes	1.941	1.785	No	No	0.516	1.513
**ZINC514290392**	No	0.439	No	No	2.482	2.185	No	No	0.285	1.307
**Efavirenz**	No	-0.429	No	No	3.035	1.4	Yes	No	1.924	0.439
**Etravirine**	No	0.006	No	Yes	2.783	0.701	Yes	No	0.320	0.986
**Rilpivirine**	No	-0.196	No	Yes	2.588	0.731	Yes	No	0.472	1.194
**Doravirine**	No	0.578	No	No	2.502	2.094	Yes	No	0.307	2.853

[a]A compound with a positive values of AMES mutagenicity test is mutagenic and therefore may act as a carcinogen; A hERG [b]I/[c]II inhibitors could cause the development of the acquired long QT syndrome, which leads to fatal ventricular arrhythmia; [d]A compound with positive tests could be associated with disrupted normal function of the liver; [e]A compound with positive tests could have a high potential adverse effect for products applied to the skin, e.g. cosmetics and antifungals; [m]measured in log mg/kg/day. If value is ≤ 0.477 log mg/kg/day is considered to be low, while > 0.477 log mg/kg/day is considered to be high; [g]measured in mol/kg. [h]measured in log mg/kg_bw/day; [i]measured in log μg/L. If value is< -0.5 log μg/L is considered to be toxic; [j]measured in log mM. If log LC50values < -0.3 indicate high acute toxicity

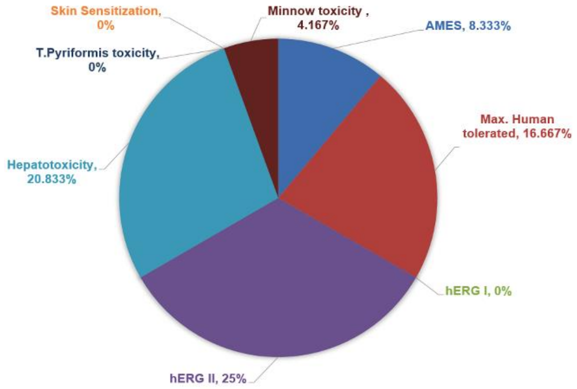

Figure S3. Pie chart representation of the alerts distribution for selected NPs

Table S8. Approved drugs, ZINC NPs and their predicted antiviral activity (IC_{50} and procent inhibition) against HIV-1 RT using HIVprotI online platform (bioinfo.imtech.res.in/manojk/hivproti/)

Name	inhibition, %	Predicted IC_{50}, μM
Doravirine	45.47	31.34
**ZINC2103242**	**52.47**	**35.58**
ZINC514290392	49.43	30.85
Etravirine	50.51	43.51
ZINC142491991	49.98	47.4
ZINC142492210	39.12	47.4
ZINC238760037	46.4	18.97
ZINC33975071	43.63	18.92
**ZINC37538901**	**47.74**	**21.48**
**ZINC38321654**	**41.55**	**25.59**
ZINC38321675	37.96	16.91
ZINC40879757	50.58	26.23
**ZINC67912677**	**27.65**	**13.26**
ZINC70455365	43.55	18.87
ZINC72320065	42.42	24.63
ZINC95486141	45.87	50.99
ZINC95911423	39.84	13.15
ZINC95911489	40.16	28.78
ZINC96023826	48.48	30.57
ZINC96113160	47.74	31.34
ZINC96113204	49.93	14.02
ZINC96269030	38.97	38.92
Efavirenz	50.5	-
ZINC4340567	49.29	2
ZINC771797	44.23	99.86
Rilpivirine	50.46	31.83

ZINC77265897	39.12	37.74
ZINC976902	46.45	38.07

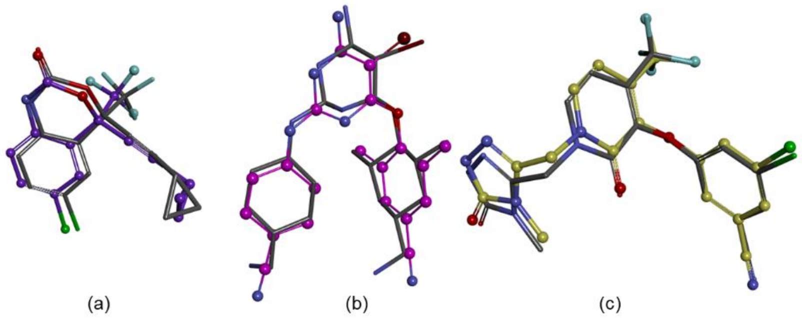

Figure S4. Efavirenz (RX-purple; the docked pose–grey)(a), Etravirine (RX-magenta; the docked pose –grey) (b), and Doravirine (RX-yelow; the docked pose–grey) (c)

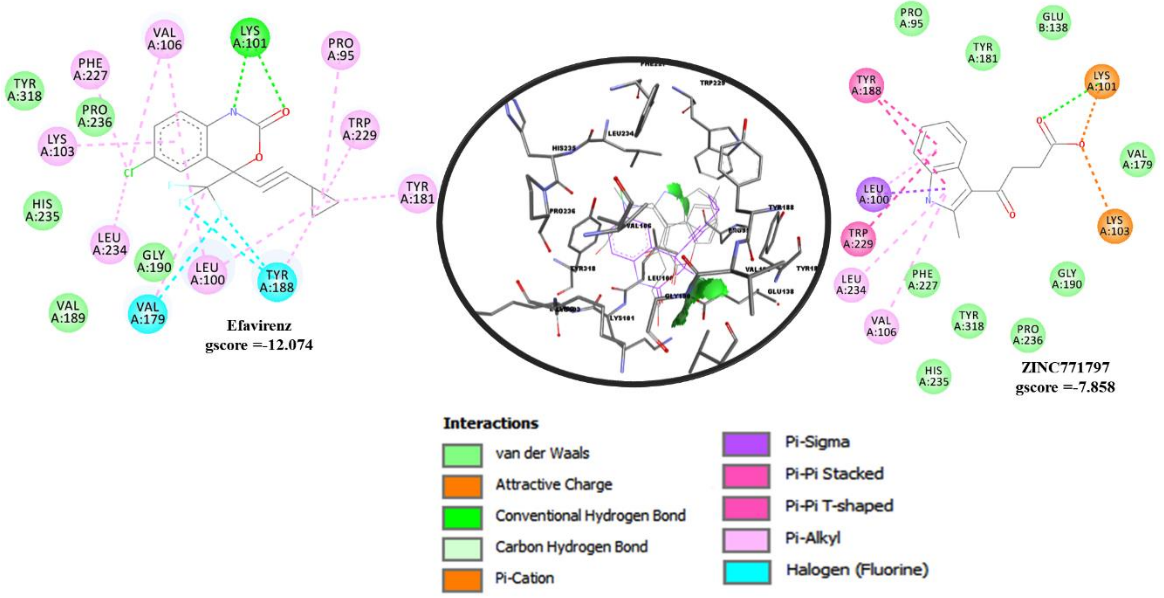

Figure S5. The selected docked NP (non-toxic) in the 1FK9 binding site – efavrinez and ZINC771797 (2D presentation); in the circle, the efavrinez (purple) and the selected NPs in 1FK9 are presented (3D variant); the green color indicates de hydrogen bonding area

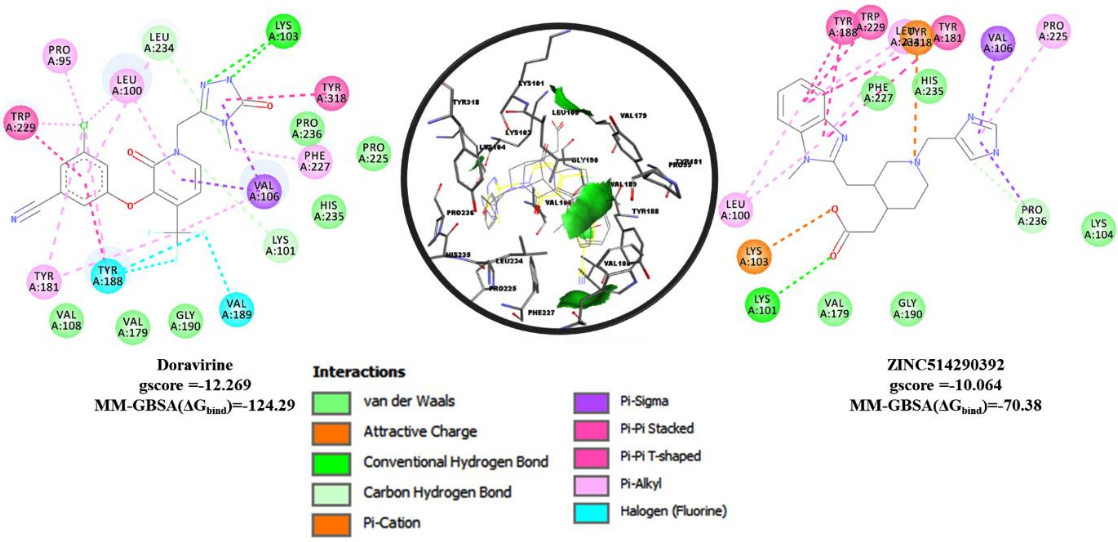

Figure S6. The selected docked NP in the 4NCG binding site – doravirine and ZINC514290392 - 2D presentation); in the circle, the doravirine (yelow) and the selected NPs in 4NCG are presented (3D variant); the green color indicates de hydrogen bonding area

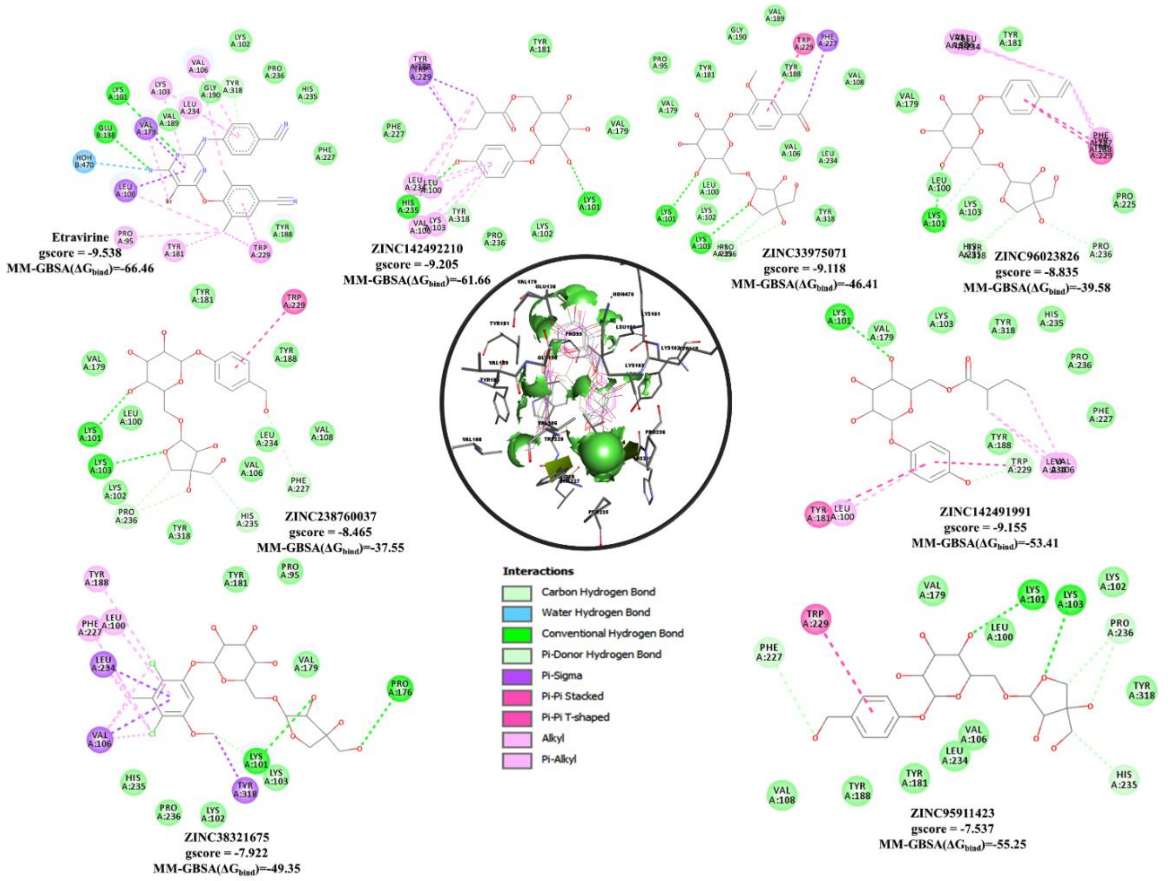

Figure S7. The selected docked NP (non-toxic) in the 3MEC binding site (2D presentation); in the circle, the etravirine (magenta) and the selected NPs in 3MEC are presented (3D variant); the green color indicates de hydrogen bonding area; MM-GBSA(ΔG_{bind})is expressed in kcal/mol

Table S9. Docked interaction analysis of selected NPs with target proteins 3MEC and 4NCG

	Bonds length in Å	Category	Type	Active site residues	NP:Atoms
ZINC37538901	2.745	Water Hydrogen Bond; Conventional Hydrogen Bond	Hydrogen Bond	B:HOH470:O	ZINC37538901:O21
	2.763	Conventional Hydrogen Bond	Hydrogen Bond	A:TYR188:O	ZINC37538901:O25
	2.657	Conventional Hydrogen Bond	Hydrogen Bond	A:LYS101:N	ZINC37538901:O21
	2.801	Conventional Hydrogen Bond	Hydrogen Bond	A:LYS101:NZ	ZINC37538901:O20
	2.958	Conventional Hydrogen Bond	Hydrogen Bond	A:TYR181:N	ZINC37538901:O25
	3.625	Pi-Sigma	Hydrophobic	A:VAL106:CG2	ZINC37538901
	3.835	Pi-Sigma	Hydrophobic	A:LEU234:CD1	ZINC37538901
	3.089	Salt Bridge	Hydrogen Bond;Electrostatic	A:LYS103:NZ	ZINC37538901:O24
	4.331	Attractive Charge	Electrostatic	A:LYS101:NZ	ZINC37538901:O24
	4.630	Alkyl	Hydrophobic	A:LYS103	ZINC37538901:C14
	5.405	Alkyl	Hydrophobic	A:VAL106	ZINC37538901:C14
	4.641	Alkyl	Hydrophobic	A:LEU234	ZINC37538901:C15
	4.557	Alkyl	Hydrophobic	A:VAL106	ZINC37538901:C16
	4.785	Alkyl	Hydrophobic	A:LEU234	ZINC37538901:C16
	5.043	Pi-Alkyl	Hydrophobic	A:TYR188	ZINC37538901:C15
	5.406	Pi-Alkyl	Hydrophobic	A:TYR188	ZINC37538901:C16
	4.537	Pi-Alkyl	Hydrophobic	A:PHE227	ZINC37538901:C16
	4.122	Pi-Alkyl	Hydrophobic	A:TRP229	ZINC37538901:C15
	4.786	Pi-Alkyl	Hydrophobic	A:TRP229	ZINC37538901:C15
	4.347	Pi-Alkyl	Hydrophobic	A:TYR318	ZINC37538901:C14
ZINC38321654	3.328	Water Hydrogen Bond;Conventional Hydrogen Bond	Hydrogen Bond	B:HOH470:O	ZINC38321654:O23
	2.704	Conventional Hydrogen Bond	Hydrogen Bond	A:LYS101:N	ZINC38321654:O23
	3.305	Conventional Hydrogen Bond	Hydrogen Bond	A:LYS103:N	ZINC38321654:O25
	2.816	Conventional Hydrogen Bond	Hydrogen Bond	A:TYR318:OH	ZINC38321654:O26
	2.694	Conventional Hydrogen Bond	Hydrogen Bond	B:GLU138:OE1	ZINC38321654:O22
	3.120	Conventional Hydrogen Bond	Hydrogen Bond	A:PRO236:O	ZINC38321654:O25
	2.445	Conventional Hydrogen Bond	Hydrogen Bond	A:HIS235:O	ZINC38321654:O26
	3.164	Carbon Hydrogen Bond	Hydrogen Bond	A:LYS101:O	ZINC38321654:C17
	4.029	Pi-Donor Hydrogen Bond	Hydrogen Bond	A:TYR318	ZINC38321654:O25
	3.259	Pi-Sigma	Hydrophobic	A:TRP229	ZINC38321654:C16
	5.374	Pi-Pi T-shaped	Hydrophobic	A:TRP229	ZINC38321654
	5.925	Pi-Pi T-shaped	Hydrophobic	A:TRP229	ZINC38321654
	5.424	Pi-Alkyl	Hydrophobic	A:VAL106	ZINC38321654

ZINC67912677	3.158	Water Hydrogen Bond;Conventional Hydrogen Bond	Hydrogen Bond	B:HOH470:O	ZINC67912677:O27
	2.893	Conventional Hydrogen Bond	Hydrogen Bond	A:LYS101:N	ZINC67912677:O27
	2.783	Conventional Hydrogen Bond	Hydrogen Bond	A:LYS103:N	ZINC67912677:O28
	2.643	Conventional Hydrogen Bond	Hydrogen Bond	A:HIS235:O	ZINC67912677:O25
	2.706	Conventional Hydrogen Bond	Hydrogen Bond	B:GLU138:OE1	ZINC67912677:O26
	2.686	Conventional Hydrogen Bond	Hydrogen Bond	A:LYS101:O	ZINC67912677:O28
	3.248	Conventional Hydrogen Bond	Hydrogen Bond	A:PRO236:O	ZINC67912677:O28
	3.878	Pi-Donor Hydrogen Bond	Hydrogen Bond	A:TYR318	ZINC67912677:O28
	3.446	Pi-Sigma	Hydrophobic	A:TRP229	ZINC67912677:C3
	3.639	Pi-Sigma	Hydrophobic	A:TYR181	ZINC67912677:C19
	3.840	Pi-Sigma	Hydrophobic	A:TRP229	ZINC67912677:C19
	4.779	Pi-Pi Stacked	Hydrophobic	A:TYR188	ZINC67912677
	4.820	Pi-Pi T-shaped	Hydrophobic	A:TRP229	ZINC67912677
	5.462	Pi-Pi T-shaped	Hydrophobic	A:TRP229	ZINC67912677
	4.231	Alkyl	Hydrophobic	A:VAL106	ZINC67912677:C7
	4.411	Alkyl	Hydrophobic	A:VAL108	ZINC67912677:C7
	3.135	Pi-Alkyl	Hydrophobic	A:PHE227	ZINC67912677:C7
ZINC2103242	3.125	Conventional Hydrogen Bond	Hydrogen Bond	A:TRP229	ZINC2103242:O26
	2.906	Hydrogen Bond	Carbon Hydrogen Bond	A:LYS101:O	ZINC2103242:C18
	3.871	Hydrogen Bond	Pi-Donor Hydrogen Bond	A:TRP229	ZINC2103242:O26
	3.557	Hydrophobic	Pi-Sigma	A:PRO236:CA	ZINC2103242
	5.890	Hydrophobic	Pi-Pi Stacked	A:TYR318	ZINC2103242
	4.039	Hydrophobic	Pi-Pi Stacked	A:TYR188	ZINC2103242
	5.334	Hydrophobic	Pi-Pi T-shaped	A:TRP229	ZINC2103242
	5.020	Hydrophobic	Pi-Pi T-shaped	A:TRP229	ZINC2103242
	4.844	Hydrophobic	Alkyl	A:LEU100	ZINC2103242
	4.645	Hydrophobic	Alkyl	A:VAL106	ZINC2103242
	4.186	Hydrophobic	Pi-Alkyl	A:VAL106	ZINC2103242
	5.077	Hydrophobic	Pi-Alkyl	A:PRO225	ZINC2103242
	5.383	Hydrophobic	Pi-Alkyl	A:LEU100	ZINC2103242
	4.735	Hydrophobic	Pi-Alkyl	A:LEU234	ZINC2103242
	4.849	Hydrophobic	Pi-Alkyl	A:TYR181	ZINC2103242
